# A Typology of National Park Co-management Agreements in the Era of Reconciliation in Canada

**DOI:** 10.1007/s00267-024-01997-z

**Published:** 2024-07-04

**Authors:** Kai Bruce, Monica E. Mulrennan

**Affiliations:** Department of Geography, Planning and Environment, Concordia University, Montreal, QC H3G 1M8 Canada

**Keywords:** Indigenous peoples, Protected areas, Co-management, Conservation governance, Reconciliation

## Abstract

Parks Canada, in response to commitments undertaken towards reconciliation, has signaled its readiness to reassess the participation of Indigenous peoples in the co-management of national parks, national park reserves, and national marine conservation areas (NMCAs). However, the effectiveness of co-management, as the established framework underpinning these and other longstanding partnerships between the state and Indigenous groups, has been disputed, based on an uneven track record in meeting the needs, interests, and aspirations of Indigenous communities. This paper explores the potential of co-management to facilitate reconciliation within national parks, reserves and NMCAs by developing a typology of various types of co-management agreements. Addressing a critical knowledge gap in co-management governance, we provide a comprehensive review of 23 negotiated co-management agreements involving the state and Indigenous groups in a national park context. The resulting typology categorizes these agreements according to contextual factors and governance arrangements, offering insights into the feasibility of shared governance approaches with Parks Canada. Moreover, it identifies the strengths and weaknesses of co-management agreements in fulfilling reconciliation commitments. Our findings indicate that, although Parks Canada has implemented innovative approaches to co-management and shown a willingness to support Indigenous-led conservation efforts, true shared governance with Indigenous groups, as defined by international standards, is limited by the Canadian government's evident reluctance to amend the foundational legislation to effectively share authority in national parks.

## Introduction

In 2021, Canadian Prime Minister Justin Trudeau publicly acknowledged Canada’s attempted genocide of Indigenous peoples (Canada 2021a). This admission was in response to decades of Indigenous political mobilization and the findings of transitional justice inquiries such as the Truth and Reconciliation Commission (TRC)(2015) and the National Inquiry into Missing and Murdered Indigenous Women and Girls (2019). The TRC defined reconciliation as a “mutual process to be engaged in by Indigenous and non-Indigenous peoples alike” to heal relationships through truth telling, acknowledgments, reparations, and addressing underlying structural causes to prevent future harms (Stanton 2017, p. 22). While the TRC and earlier public inquiries have provided Canada with ample guidance and principles on which to build reconciliation, progress on these actions has been slow (Yellowhead Institute 2019). The response has wavered “from serious political and socio-economic transformation to the maintenance of the status quo” punctuated, at times, by performative actions of reconciliation and redress (Henderson and Wakeham 2013, p. 9). Indeed, a host of Indigenous leaders and scholars have critiqued state-led reconciliation as a largely empty gesture that fails to address the underlying conditions that perpetuate the ongoing harms of colonialism (Coulthard 2014; Daigle 2019; Guerin 2019; Napoleon 2019; Taiaiake Alfred 2009; Whyte 2018; Wildcat et al. 2014; Wilson et al. 2019). Yet, it is recognized that, at a minimum, reconciliation will demand redress, restitution, and a rebalancing of relationships across all settler institutions (Littlechild et al. 2021). One particularly fertile area for this transformation is conservation, where growing support and recognition for Indigenous-led conservation and Indigenous Protected and Conserved Areas (IPCAs) now offer a significant opportunity to advance reconciliation (Indigenous Circle of Experts 2018; Tran et al. 2020; Zurba et al. 2019).

This “new paradigm” of conservation, characterized by support for and recognition of Indigenous forms of stewardship, carries the potential for a transformational shift in Canadian conservation bolstered by the TRC, the United Nations Declaration of the Rights of Indigenous Peoples (UNDRIP), and commitments to international biodiversity targets (Indigenous Circle of Experts 2018; Parks Canada Agency 2022b; Stevens 2014). Foundational principles and guidance in support of this revised approach were put forward by the Indigenous Circle of Experts (ICE) in the 2018 report “We Rise Together”, as part of a mandate to create recommendations for achieving Canada’s Pathway to Target 1^1^ (Indigenous Circle of Experts 2018; Nikolakis and Hotte 2019). The ICE advocated for the adoption of Indigenous frameworks for engagement, notably ethical space and two-eyed seeing, to support a shift in expectations around Indigenous-state conservation partnerships (Bartlett et al. 2012; Ermine 2007). In response, Parks Canada, the national agency responsible for national parks (including national park reserves, NMCAs, and national historic sites), has signaled its commitment to advancing Indigenous-led conservation and IPCAs through a series of recent collaborative partnerships intended to support Indigenous self-determination and reconciliation (CBC 2022). These projects reflect Parks Canada’s alignment with federal government commitments to reconciliation by “renewing nation-to-nation, government-to-government, and Inuit-Crown relationships based on recognition of rights, respect, cooperation and partnership” (Parks Canada 2019, p. 8).

While the creation of IPCAs has been a focal point of the revised approach, the dire need for redress, restitution, and reconciliation in existing protected areas is also recognized. This is particularly the case in national parks that were established as strategic tools of settler colonialism often involving the forceful dispossession or marginalization of Indigenous peoples from their lands (Johnston and Mason 2020; Townsend 2022; Youdelis 2016). Many of these Indigenous groups continue to have strained relationships with Parks Canada, prompting Parks Canada to initiate a process of offering formal apologies to those affected by the dark history of exclusion they now openly acknowledge (Dragon Smith and Grandjambe 2020; Finegan 2018; Indigenous Circle of Experts 2018). Ermineskin Cree scholar Danika Littlechild and others (2021) point out that while Parks Canada acknowledges reconciliation as a unique process specific to each Indigenous community and protected area, its mandate advances a fragmented approach that places Parks Canada rather than Indigenous people at the center of reconciliation (Littlechild et al. 2021). This is reflected in Parks Canada’s sustained commitment to shared governance as a key mechanism of transformation. The latter raises a central question; can reconciliation be achieved by enhancing “Indigenous peoples’ decision-making roles in the management of heritage places through relationship-building structures, collaborative arrangements and/or formalized cooperative management boards” – in other words, can reconciliation be achieved through co-management^2^ arrangements involving the state? (Parks Canada Agency 2022b).

The adoption by Parks Canada of co-management as an institution of governance represented a historically significant shift in approach in the 1980s, supporting a redistribution of socioeconomic benefits derived from national parks to Indigenous communities (Dearden and Bennett 2016). However, it has become a target of significant scholarly critique over time, primarily for its failure to address imbalances of power-sharing, participation, and respect of knowledge systems (e.g. Nadasdy 2003; Sandlos 2007). Many co-management structures established in national parks are legally constrained by the provisions of comprehensive land claim agreements which have limited Indigenous authority to joint advisory bodies (Fenge 1993; Sandlos 2014). Not surprisingly, the limitations of co-management’s past record of relationship-building have led some scholars to question its capacity to be repurposed to serve the ambitious promise of reconciliation. Chance Finegan (2018), a political scientist at the University of Toronto, Mississauga, has argued vociferously against the reliance of the new paradigm of protected areas in Canada on tools such as co-management. Likewise, in a recent paper focused on identifying better alternatives to a now ubiquitous co-management regime, Grey and Kuokkanen (2020, p. 920) argue that co-management “cannot be ‘tweaked to provide better outcomes for Indigenous peoples, nor can it provide a stepping stone to their self-determination”.

According to historian Mark Spence (1999), national parks are in many ways reflections of the legal and political circumstances of Indigenous people at the time they were created. The complex, piecemeal evolution of Parks Canada’s implementation of co-management arrangements in national parks, documented in detail by several scholars (Dearden and Bennett 2016; Langdon et al. 2010; Sandlos 2014), invites skepticism around co-management as an equitable and sustainable institutional arrangement for national park governance. For example, inconsistencies and tensions in Parks Canada’s approach to Indigenous engagement and collaboration have been raised in relation to politics of scale (Dearden and Bennett 2016; Timko and Satterfield 2008), inequitable policy approaches (Thomlinson and Crouch 2012), power and epistemic imbalances (Langdon et al. 2010; Lemelin and Bennett 2010; Sandlos 2014), conflicts between Indigenous and state conservation approaches (Doberstein and Devin 2004; Notzke 1995; Thomlinson and Crouch 2012), and lack of meaningful participation (Johnston and Mason 2020; Youdelis 2016). At a broader level, scholarship and policy literature indicate that Parks Canada has failed to meet the International Union for the Conservation of Nature’s (IUCN) standards for shared governance with Indigenous communities (Borrini-Feyerabend et al. 2013; Thomlinson and Crouch 2012).

Indigenous peoples around the world, including in other settler-state settings, are increasingly advancing conservation approaches that intently depart from collaborative approaches with the state (Townsend 2022). Yet, some Indigenous-led entities, including the ICE, have continued to recommend co-management as a viable governance option for Indigenous groups working to establish IPCAs or similar Indigenous-led conservation initiatives (Indigenous Circle of Experts 2018). Endorsements of co-management, while perhaps more tepid, have also been offered by conservation scholars working in contexts of Indigenous cultural resurgence. For instance, biologist Kyle Artelle, at the State University of New York, suggests that the adoption of co-management by Indigenous groups “could in turn support their agency and the resurgence of practices that have supported sustained interactions between people and places for millennia” (Artelle et al. 2019, p. 4; Indigenous Circle of Experts 2018). We mention this ongoing endorsement of co-management (whether lukewarm or enthusiastic) not to build a straw man argument, but rather to underscore the ambivalent and contradictory attitudes towards it and its significance to Indigenous self-determination and governance. Ultimately, sustainable and equitable conservation partnerships must reconcile power imbalances and respectfully engage different worldviews and identities (Dietsch et al. 2021; Moola and Roth 2019). In this sense, co-management can only support reconciliation if it enables Indigenous self-governance and self-determination through nation-to-nation relationships that go beyond mere recognition of Indigenous peoples or incorporation of Indigenous worldviews and knowledges.

Various pathways have been suggested by which co-management arrangements might evolve towards a rebalancing of power. One alternative partnership may be through co-governance or co-jurisdiction, implying shared control and decision-making, which is increasingly discussed in northern and western Canadian co-management contexts (Clark and Joe-Strack 2017; Martin 2016; Ottawa 1992; Simms et al. 2016). Co-jurisdiction was also a recommendation of the RCAP for environmental management (Royal Commission on Aboriginal Peoples 1996, p. 6). Sandlos (2014) suggests a more radical approach to co-management that involves a devolution of power to local Indigenous stewardship regimes. These and related alternatives uphold what Dale Turner, a Temagami First Nation scholar of Indigenous politics, terms constitutional and political justice, wherein Indigenous laws and culture stand on their own authority (Turner 2013).

Following Townsend (2022), whose recent research explored various pathways to reconciliation through IPCAs, we argue that the extent to which co-management can support Indigenous governance and self-determination – through power-sharing arrangements – is critical to its capacity to fulfill commitments to reconciliation (Reed et al. 2021; Royal Commission on Aboriginal Peoples 1996). Despite the attention given to power inequities, most critiques of co-management are informed by case-study analysis rather than a more comprehensive examination of a fuller range of co-management experiences, each of which is “influenced by the history and legacy of the older systems, its power relations and conflict levels” (Petursson and Kristofersson 2021, p. 4). To address this, this paper aims to recognize and reveal differences and commonalities across a range of distinct co-management types. We apply a typology approach, focussed on contextual factors and governance considerations, to a comprehensive review of co-management agreements negotiated between Indigenous groups and Parks Canada. This not only aids in unraveling and comprehending the potential that co-management, as a wide-ranging governance institution, offers, but it also supports a descriptive narrative and a lens for delving deeper into the mechanisms (e.g. board composition, dispute resolution) available to support renewed relationships and reconciliation (Hill et al. 2012).

According to the Canadian National Parks Act (2000), co-management is achievable only through negotiated agreements and contracts, but is not necessarily guaranteed to Indigenous groups (Dearden and Bennett 2016). As there is no published scholarship on the specificities of particular agreements, our objective was to address this gap through a comprehensive typology of the spectrum of co-management agreements in national parks, national park reserves, and NMCAs^3^. Comparative assessments of national park co-management agreements in the literature are few, and almost invariably based on individual or sample-based case studies (e.g. Martin 2016; Thomlinson and Crouch 2012; Timko and Satterfield 2008). Based on these studies, claims-based management in the Canadian north and west has been shown to define roles for Indigenous people in planning and management while parks in the south lag behind in power-sharing. However, no systematic description or comparison between arrangements exists to date. Finally, many existing studies pre-date Parks Canada’s evolved commitments to engaging with Indigenous people as well as the establishment of several new parks. This study addresses these knowledge gaps and builds upon a preliminary typology developed by Milko (2020) as well as an internal policy review of co-management agreements of Parks Canada’s (2018).

The paper begins with a review of literature addressing the evolution of perspectives and expectations around co-management. We then describe the methods we used to conduct our review co-management agreements across the national park, national park reserve, and NMCA network^4^ and to develop a typology of these agreements. We then apply the resulting typology to an examination of how co-management agreement types align with the IUCN’s shared governance standards based on a typology of governance options developed by Milko (2020), building from the co-management typologies of Hill et al. (2012) and Lyver et al. (2014). We conclude with a general discussion addressing the contribution of our findings to existing scholarship, and some implications for reconciliation.

## Literature Review

Two challenges characterize the literature on co-management: wavering adoption by government and a longstanding academic debate (Clark and Joe-Strack 2017). This review focusses on key studies addressing the changing roles and assessment of co-management institutions over time. We privilege case studies and collaborative research conducted with Indigenous communities across Indigenous Canada/Turtle Island and other settler states such as Aotearoa/New Zealand and Australia. Additionally, we provide a general overview of approaches to understanding the governance elements of co-management and power-sharing arrangements in governance.

### Co-Management and Evolving Expectations for Collaboration in Conservation

Since the 1970s, attitudes towards and understandings of co-management have evolved significantly. Despite vague definitions and contested assessments, co-management has become ubiquitous across environmental management contexts and government policy, especially in northern Canada (Gray and Kuokkanen 2020). Various top-down and community-driven factors inform the particularities of each co-management arrangement (Notzke 1995). Comprehensive land claims or modern treaties, which drove the establishment of several northern national parks, provided the initial impetus for co-management arrangements (Atkinson 2001; Notzke 1995). Co-management was introduced as a compromise under land claims negotiations when Indigenous and customary jurisdictions conflicted with the interests of the state (Gray and Kuokkanen 2020; Pasternak 2017; Mulrennan and Scott 2005). Twenty years ago when examples of co-management were limited, co-management under constitutionally-protected land claims was viewed as a “permanent, institutionalized relationship” between Indigenous groups and the state (Mulrennan and Scott 2005; Usher 1996, p. 1). Co-management was wielded as an “instrument of public government”, often presented by the state as the sole alternative available to Indigenous groups, in often fraught contexts of state-sanctioned resource development (Mulrennan and Scott 2005; Usher 1993, p. 6). As co-management became more ubiquitous, researchers began to observe the strategic uptake of co-management by Indigenous communities as a mechanism for their engagement with the state (Berkes 2009; Diver 2016; Hill et al. 2012; Lyver et al. 2014; Martin 2016; Mulrennan and Scott 2005; Zurba et al. 2012). Yet, as case studies attest, the state and Indigenous leaders often interpret the purpose of co-management differently, with Indigenous groups aspiring to arrangements closer to co-jurisdiction or co-governance while the state focused on consultative arrangements (Martin 2016; Parsons et al. 2021). The strategic uptake of co-management by Indigenous governments as a tool of engagement with the state and its attendant outcomes, are dependent upon the legal and constitutional tools available to Indigenous groups at the time (Mulrennan and Scott 2005). Indeed, the framework for recognition of Indigenous rights under *Constitution Act*, 1982 s. 35 has led to a tangled web of jurisprudence that creates unequal opportunities for Indigenous groups to engage in co-management (Nikolakis and Hotte 2019). Furthermore, the courts have tended to avoid interpretations of Indigenous rights that conflict with Canada’s underlying assertion of sovereignty and title over Indigenous territories, thereby limiting the prospect of co-jurisdiction (Borrows 2015).

Amid shifting perspectives regarding co-management from both Indigenous and state authorities, co-management has also sparked contentious debates within academic circles. In its early form, it was viewed as a top-down policy solution to devolve and share power and responsibilities with local level management systems (Berkes 1987; Feit 1988; Usher 1993). In response, critiques during the 1980s and 1990s tended to focus on the power asymmetries of co-management, particularly its capacity to co-opt and displace Indigenous governance and knowledge systems, the very dynamics it also purported to remediate (Berkes et al. 1991; Nadasdy 2003; Stevenson 2006). Case studies from this era suggested that co-management institutions had little to offer Indigenous communities with respect to self-determination (Rodon 1998). As opinions shifted during the 2000s, co-management scholars began to re-consider co-management as a step-wise relationship-building process through which more equitable levels of power-sharing, participation, and knowledge integration could be achieved (Berkes 2009; Carlsson and Berkes 2005; Natcher et al. 2005). Feit (2005), informed by Cree perspectives, argued that co-management regimes may in fact enable co-governance and Indigenous autonomy despite possibly contradictory intentions of the state. Meanwhile others have proposed that co-management be re-tooled to center community objectives and focus on reconciliation and redress (Armitage et al. 2020; Jacobson et al. 2016). Despite these calls for change, power imbalances continue to define the experience of many Indigenous groups with co-management arrangements in Canada (Sandlos 2014; White 2018; Youdelis et al. 2020).

Another trend that has received wide-spread support, particularly from Indigenous scholars and community leaders, has been the search for solutions to the limitations of co-management. The potential of Indigenous frameworks of engagement, notably ethical space (Ermine 2007), Two-Eyed Seeing/Etuaptmumk (Bartlett et al. 2012), or two-row wampum/Kaswentha (McGregor 2002), has encouraged renewed expectations for Indigenous-state relationships in various contexts including conservation governance (Indigenous Circle of Experts 2018; Laurila 2019; Littlechild and Sutherland 2021; Nikolakis and Hotte 2021; Zurba et al. 2021). This shift in evolving expectations is reinforced by the momentum established by conservation organizations at various scales, which have outlined principles and provided guidance for restructuring conservation approaches in line with Canada’s acknowledgment of Indigenous rights to self-determination and self-government, and its commitment to the renewal of relationships (Moola and Roth 2019; M’sɨt No’kmaq et al. 2021). Along with the 2018 ICE report, the United Nations Declaration on the Rights of Indigenous Peoples (UNDRIP), initially endorsed by Canada in 2016 in response to the TRC, and subsequently implemented in 2021, reaffirms the right of Indigenous peoples to conserve, manage, and sustainably utilize their lands and territories (M’sɨt No’kmaq et al. 2021; United Nations 2007). Similar calls to support Indigenous leadership in conservation have been included in international sustainability agreements since the 1987 *Brundtland Report* (Higgins 1998).

While Indigenous frameworks of engagement offer the potential to improve co-management arrangements to better address Indigenous priorities and needs, a challenge persists in assessing the effectiveness of co-management for fulfilling such objectives, given its diverse range of implementations and the varying contexts in which these are situated (Clark and Joe-Strack 2017). Various governance analysis frameworks and typologies have been developed to foster understanding of the diversity of power-sharing arrangements in co-management. For instance, frameworks for studying governance structures exist that consider the dynamics and interplay between scale, context, institutions, actors, and actions (Ostrom 2005; Petursson and Kristofersson 2021; Schröter et al. 2014; Young 2002). Typologies have been employed to analyze collaborative arrangements in protected areas, wildlife, and natural resource management, assessing degrees of power-sharing and participation through various parameters and metrics. Among these, the agreements themselves may serve as a parameter for analysis (Hill et al. 2012; Hughey et al. 2017; Plummer and Fitzgibbon 2004; Puley and Charles 2022; Sen and Nielsen 1996; Wyatt et al. 2013). This includes two typology studies of co-management within national parks in settler state settings, neither of which have addressed the Canadian context (Lyver et al. 2014; Martin 2016). Typology approaches are typically based in either or both rational choice institutionalism and sociological institutionalism (Hill et al. 2012; Plummer and Fitzgibbon 2004). The former, which assumes “that actors behave as utility maximizers to rank their priorities within institutional constraints” (Hill et al. 2012, p. 23) is often complemented by the latter, which assumes social and political contexts influence actors’ calculations of utility and considers the extent to which institutions “enable and empower, provide licenses, and create opportunities” (Jentoft et al. 1998, p. 427). In light of these methodological considerations, the typology approach serves as an insightful but limited analytical tool for assessing the potential of co-management to support Indigenous-state reconciliation, as relational aspects and grounded perspectives have been largely set aside. While we by no means wish to underplay the centrality of grounded relationships in reconciliation, we argue that the limitations of the chosen approach are justifiable given the increasingly conflicted understandings of co-management and its potential for supporting reconciliation at a legal governance level.

## Methods

### Identifying Agreement Cases

The creation of the co-management agreement typology involved several iterative stages of data collection and analysis. As a first step, the primary author identified relevant agreements in order to prepare a database; that is, negotiated agreements that explicitly aim to establish cooperative approaches to national park, national park reserve, or NMCA management between an Indigenous group and Parks Canada. As such, contribution agreements and pre-establishment agreements were excluded. Since the objective was to establish an overall appreciation of co-management, the total population of agreements was reviewed rather than a sample or case-study approach. This included the incorporation of agreements from the Canadian south and Atlantic regions as well as historic treaty contexts. To identify existing agreements, the primary author reviewed journal articles, plans, reports, media releases, and news articles. Through this process, 27 individual national parks and 4 NMCAs were identified, which involved Indigenous peoples entering into cooperative management agreements. Direct contact was then made with Indigenous governments and park managers by email and phone to request access to the agreements. Ultimately, the primary author reviewed 23 cooperative agreements across the national park and NMCA system^5^ published between 1984 and 2022, two of which are reviewed based on data extracted from secondary sources (i.e., journal articles, reports, media releases, published plans, or web pages). These agreements correspond to cooperative management arrangements of 21 national parks and 3 NMCAs. Additional relevant agreements identified in the review were excluded from the study for diverse reasons: inaccessibility either due to confidentiality issues, difficulties encountered in contacting park representatives, or lack of secondary data. Table 1 presents a list of agreements and contextual data organized by the year of agreement. “Assignments” of agreements to the agreement typology, explained in the following section, are best understood as alignments, as agreements may have been amended since their date of origin.

**Table 1:** List of agreements, year of agreement, year of park establishment, province/territory of park, Indigenous representation, legal/jurisdictional context, ordered by year of agreement

Agreement	Agreement Year	Park Est.	Province/Territory	Indigenous Groups	Legal context	Type^a^
The Inuvialuit Final Agreement (Ivvavik NP)	*1984*	*1984*	YT	Inuvialuit	Inuvialuit Final Agreement (1984)	CMB
KFN Final Agreement, CAFN Final Agreement (Kluane NP and NPR)	*1993, 2004*	*1976*	YT	Kluane First Nation and Champagne and Aishihik First Nation	Kluane First Nation (KFN) Final Agreement (2004), Champagne and Aishihik First Nations (CAFN) Final Agreement (1993), Umbrella Final Agreement (1990)	CMB
An Agreement for the Establishment of a National Park on Banks Island (Aulavik NP)	*1992*	*1992*	N.W.T.	Inuvialuit	Inuvialuit Final Agreement (1984)	CMB
Vuntut Gwitchin First Nation Final Agreement (Vuntut NP)	1992	1993	YT	Vuntut Gwitchin First Nation	Vuntut Gwitchin First Nation (VGFN) Final Agreement (1993), Umbrella Final Agreement (1990)	CMB
Gwaii Haanas Agreement, (Gwaii Haanas Marine Agreement*) (Gwaii Haanas NPR and NMCA)	*1993 (2010)*	*1993 (1988)*	B.C.	Haida Nation	Active Claim, Court Decisions (Haida Nation v. British Columbia (2004))	CMA
Federal-Provincial Memorandum of Agreement for Wapusk National Park	*1996*	*1996*	MB	Fox Lake First Nation and York Factory First Nation	Treaty 5 (1875), Northern Flood Agreement (1997)	CMB
Agreement to Establish a National Park in the Inuvialuit Settlement Region Near Paulatuk, NWT, (IBA for Park Expansion) (Tuktut Nogait NP)	*1998 (2005)*	*1998*	N.W.T.	Inuvialuit (Inuvialuit Game Council, Inuvialuit Regional Corporation, Paulatuk Community Corporation, Paulatuk Hunters and Trappers Committee)	Inuvialuit Final Agreement (1984), Sahtu Dene and Metis Comprehensive Land Claim (1994), Treaty 11 (1921)	CMB
Terms of Agreement for Senior Management Forum (Riding Mountain NP)	*1998*	*1933*	MB	Keeseekoowenin Ojibway First Nation	Treaty 2 (1871), Keeseekoowenin Ojibway First Nation Specific Claim of 1906 (Indian Claims Commission, 2005)	ABA
Inuit IBA for Auyuittuq, Quttinirpaaq and Sirmilik National Parks	*1999*	*1972, 1988, 2001*	NT	Qikiqtani Inuit Association	Nunavut Land Claims Agreement (1993)	CMB
Nahanni National Park Reserve Interim Park Management Arrangement	*2003 (2009)*	*1976*	N.W.T.	Dehcho First Nations (represented by Deh Cho First Nations Grand Chief)	Treaty 11 (1921), Treaty 8 (1899), Deh Cho First Nations Framework Agreement (2001)	CMB
An Inuit Impact and Benefit Agreement for Ukkusiksalik National Park of Canada	*2001*	*2003 (2014)*	NT	Kivalliq Inuit Association representing the Inuit of Nunavut Settlement Area	Nunavut Land Claims Agreement (1993)	CMB
Inuit Impact and Benefit Agreement for Tongait KakKasuangita SilakKijapvinga/Torngat Mountains NPR	*2005*	*2008*	N.L.	Inuit Labrador (Labrador Inuit Association) and Nunavik Inuit (Makivik Corporation)	Labrador and Inuit Land Claims Agreement (2005), Nunavik Inuit Land Claims Agreement (2007)	CMB
Riding Mountain Forum Agreement (Terms of Agreement)	*2006*	*1933*	MB	Coalition representing Keeseekoowenin Ojibway First Nation, Ebb and Flow First Nation, Waywayseecappo First Nation, Rolling River First Nation, Tootinaowaziibeeng First Nation, Gambler First Nation, and Sandy Bay Ojibway First Nation	Treaty 2 (1871), Keeseekoowenin Ojibway First Nation Specific Claims of 1906 (Indian Claims Commission 2005)	ABA
Agreement for the cooperation in the planning and management of Pacific Rim National Park Reserve (2006) (to become Maa-nulth Treaty Side Agreement)	*2006 (2012)*	*1970*	B.C.	Huu-ay-aht First Nations, Toquaht Nation, Uchuckleshaht Tribe, and Ucluelet First Nation	Maa-nulth First Nations Final Agreement (not ratified until 2011)	CMB
Memorandum of Cooperation between Caldwell First Nation and Walpole Island First Nation and Point Pelee National Park	2011	1918	ON	Caldwell First Nation and Walpole Island First Nation	Southern Ontario Treaties (1764–1862) (Treaty 2, 1871)	RBA
Terms of Reference for Point Pelee National Park First Nations Advisory Circle	2011	1918	ON	Caldwell First Nation and Walpole Island First Nation	Southern Ontario Treaties (1764–1862) (Treaty 2, 1790)	ABA
PC Interim Arrangement (Kejimkujik and Cape Bretons Highlands NP)	*2012 (2017)*	*1967*	N.S.	Mi’kmaq of Nova Scotia as represented by the Assembly of Nova Scotia Mi’kmaq Chiefs (13 at time of signing, but may fluctuate)	Peace and Friendship Treaties (1725–1779), TOR for a Mi’kmaq-Nova Scotia-Canada Consultation Process	RBA
Inuit Impact and Benefit Agreement for Qausuittuq NP	*2015*	*2015*	NT	Qikiqtani Inuit Association representing the Inuit of Qikiqtani region and of Resolute, NT in particular	Nunavut Land Claims Agreement (1993)	CMB
Establishment Agreement between the Government of Canada and the Łutsël K’é Dene First Nation (Thaidene Nëné NPR)	*2019*	*2019*	N.W.T.	Łutsël K’é Dene First Nation	Treaty 8 (1899), Land Claims and self-Governance negotiations for both Akaitcho Dene First Nations and Northwest Territory Métis Nation	CMA
Tallurutiup Imanga NMCA Inuit Impact and Benefit Agreement	*2019*	*TBD*	NT	Qikiqtani Inuit Association	Nunavut Land Claims Agreement (1993)	CMA
Memorandum of Understanding - PCA and Saugeen Ojibway First Nation (Bruce NP and Fathom Five NMA)	*2021*	*1987*	ON	Saugeen Ojibway First Nation	Southern Ontario Treaties (1764–1862) (Treaty 72, 1854), R. v. Jones and Nadjiwon [1993] (Blair, 1996), On-going legal claims on treaty and title	RBA
Ndahecho gondié gháádé Agreement (Nahʔą Dehé/Nahanni NPR)	*2022*	*1976*	N.W.T.	Nahʔą Dehé Dene Band and Dehcho First Nations	Treaty 11 (1921), Treaty 8 (1899), Deh Cho First Nations Framework Agreement (2001)	CMA

^a^See Table 2 for agreement types: *RBA* Relationship-Building Agreement, *ABA* Interest-based Advisory Body Agreement, *CMB* Co-management Board Agreement, *CMA* Consensus Management Agreement

*Gwaii Haanas Agreement and the Gwaii Haanas Marine Agreement are herein considered as one agreement as the latter primarily extends the roles and responsibilities of the former to the Gwaii Haanas Marine Area

### Agreement Database

As a second step, the primary author scanned the content of agreements to create a database which involved coding each agreement according to a set of parameters. For the initial agreement scan, these were selected based on parameters applied in established governance typologies that were potentially relevant to negotiated agreements (Borrini-Feyerabend 1996; Bowie 2013; Boyd and Lorefice 2018; Hill et al. 2012; Hughey et al. 2017; Lyver et al. 2014; Sen and Nielsen 1996; Wyatt et al. 2013). Contextual factors were also deemed important for supporting comparisons between negotiated agreements in environmental and resource management involving Indigenous groups and the state (Caine and Krogman 2010; O’Faircheallaigh et al. 2003; Petursson and Kristofersson 2021). Thus, the initial databasing of agreements included the following parameters, among others: history of park establishment; treaty context; transparency; legal enforceability; Indigenous involvement and representation; harvest rights; capacity-building; funding; economic opportunities; governance structures; time-scale; respect and control of Indigenous Knowledge(s), language, and heritage; principles of Indigenous governance; and intercultural purpose.

### Typology

The third step, to create a governance typology of the co-management agreements, involved narrowing parameters from the agreement database to a common set of differentiable dimensions and subparameters relating to context and governance. Following Mitchell and Shortell (2000), this selection method took an iterative approach to categorization and revision. This involved revisiting agreements to validate certain subparameters, and then adjusting subparameters to fit the database, and so forth. This process was necessary because the initial set of parameters of the agreement database covered a wide array of themes that in many cases exceeded the scope of content provided by the negotiated agreements and in other cases did not produce meaningful results. For example, despite the relevance of parameters addressing provisions relating to Indigenous governance, Indigenous worldviews, Indigenous knowledges, or cultural values in co-management, the diversity and complexity of these considerations, both between agreements and within agreement types, limited any meaningful analysis. The practice of redacting politically and culturally sensitive material from public agreements further limited the use of this type of parameter. Given these constraints, including the impossibility of determining which agreements had been redacted, we opted to focus more narrowly on contextual governance parameters which were available for all agreements. While this permitted the creation of a typology, the excluded parameters limit the potential of the study in accounting for certain aspects of agreements that are perhaps more reflective of the priorities of Indigenous communities. The final iteration of the typology employs four co-management agreement types under Parks Canada, distinguished by two primary dimensions of context and governance which correspond to the following contextual and governance subparameters.

1. Context: Time of agreement implementation relative to park establishment date (proxy for early involvement of Indigenous peoples) (Boyd and Lorefice 2018; Reo et al. 2017; Sen and Nielsen 1996); reason for Indigenous involvement (Boyd and Lorefice 2018); legal context (Wyatt et al. 2013), geographical context (Petursson and Kristofersson 2021)
2. Governance: legal strength (Hughey et al. 2017), Indigenous representation (Hughey et al. 2017; Petursson and Kristofersson 2021); governance body composition, mandate, and decision-making processes (Gibson et al. 2020; Hughey et al. 2017; Wyatt et al. 2013); processes of adaptation (Hughey et al. 2017)

Borrowing from Parks Canada’s own nomenclature^6^ for co-management arrangements (2018), these are referred to herein as: relationship-building agreements (RBA), interest-based advisory body agreements (ABA), cooperative management agreements (CMB), and consensus management agreements (CMA). Key differences across context and governance dimensions between agreements are presented in Table 2. In combination with the agreements reviewed, we explore key differences and illustrative examples between agreement types along the context and governance dimensions established in the typology, presented in Tables 3 and 4.

**Table 2:** Summary of key differences across four co-management agreement types distinguished by context and governance parameters

			Agreement Type			
			Relationship-Building (RBA) (*n* = 3)	Interest-based Advisory Body (ABA) (*n* = 3)	Cooperative Management Board (CMB) (*n* = 12)	Consensus Management (CMA) (*n* = 5)
		Agreement Examples	Memorandum of Cooperation, MOU	Forum, MOU, TOR	Establishment Agreements, IIBAs, Modern Treaties	Establishment Agreements, IBAs
Context	Indigenous Involvement	Park Establishment			✓	✓
		Post- Park Establishment	✓	✓	✓	✓
	Reason for Involvement	Renewing Relationships	✓	✓		✓
		Legal Requirements			✓	✓
	Legal Context	Historic Treaty	✓	✓		✓
		Asserted Claims	✓	✓		✓
		Settled Claim or Self-Governance Agreements			✓	✓
	Geographical Context	General region; remoteness	South/Atlantic; Park near urban centers	South/Atlantic; Park near urban centers	North/Pacific Coast; Park is remote	North/Pacific Coast; Park is remote
Governance	Number of Indigenous groups represented	Static	✓	✓	✓	✓
		Allows for withdrawal alone			✓	
		Flexible Indigenous representation	✓	✓		
	Legal Strength	Legally-Binding		✓/✗	✓	✓
	Governance Body Composition	Not Defined	✓	✓	✓	
		Parks Canada/Crown government outnumber Indigenous appointees			✓	
		Indigenous outnumber Parks Canada/Crown appointees		✓	✓	
		Equal or Greater Indigenous Representation with Independent Chair or non-voting senior representative			✓	✓
	Role of Appointees/Members	To represent interests of parties	✓	✓	✓	✓
		To represent public interest			✓	✓
	Governance Body Mandate	Recommendations based on discussion between parties provided to Minister		✓		
		Recommendations by majority rule *and/or* consensus between parties provided to Minister		✓	✓	
		Recommendations based on consensus discussion between parties provided to Minister and Indigenous Leadership			✓	✓
	Disagreement, Issue, Dispute Resolution	Undefined	✓	✓		
		Mediation Processes		✓	✓	✓
		Arbitration Processes			✓	✓
		By consensus, only Minister’s authorities retained		✓	✓	
		By consensus, authorities of all parties retained				✓
	Amendment Provisions	Via Mutual written Consent between parties	✓	✓	✓	✓
		Via external mechanisms			✓	
	Termination of Agreement	Undefined			✓	
		Unilateral	✓			✓
		By Mutual Agreement		✓	✓	✓
		Cyclical		✓	✓/✗	✓
	Implementation Review	Undefined	✓		✓	
		Included	✓	✓	✓	✓
	Agreement Review	Undefined	✓		✓	
		Frequent (i.e. annual)	✓			
		Less Frequent (i.e. 5-10 years)			✓	✓

**Table 3:** Illustrative examples of differences between agreements according to the context parameters of the typology

Context parameter	Difference	Illustrative example
Early Involvement and Reasons	**ABA**: Agreement negotiated post-park establishment	Riding Mountain NP was established in 1933. The Senior Officials Forum Agreement 1996 was implemented in recognition of past disharmony and mistrust. Keeseekoowenin First Nation was evicted in land expropriation in 1935 (Indian Claims Commission 2005)
	**CMB**: Agreement negotiated for park establishment	Vuntut NP was established through the VGFN Final Agreement in 1993. Agreement preamble recognizes shared objectives for the establishment and management of park.
	**CMB:** Agreement negotiated post-park establishment	The IIBA for Auyuittuq, Quttinirpaaq and Sirmilik National Parks (1999) subsumed Baffin Island National Park Reserve into the NLCA (becoming Auyuittuq National Park)
	**CMA**: Agreement negotiated post- park establishment	Nahanni NPR was established in 1976 without consent of local First Nations. The Ndahecho Gondié Gháádé Agreement (2022) was signed in 2022 in recognition of lack of involvement of local First Nations in establishment and management of park.
Legal Context	**RBA**: Historic Treaty and Asserted Claims	Bruce NP is within Southern Ontario treaty region (1974–1892). There are two on-going legal claims on SON’s behalf relating to treaty and title
	**CMB:** Historic Treaty and Asserted Claims	The Federal-Provincial Memorandum of Agreement for Wapusk National Park (1996) recognizes Fox Lakes’ active specific claim at the time and is based in Treaty 5
	**CMA**: Asserted Claims and Court Decisions	The Haida Nation and Canada have “agreed to disagree” regarding unresolved title and divergent views of sovereignty (1993).
	**CMA**: Self-Governance Negotiations and Historic Treaty	Thaidene Nëné is based in historic treaty context where self-governance negotiations are underway through the Akaitcho Process. The Thaidene Nëné Agreements are described as an implementation mechanism of Treaty 8 (1899) (Łutsel K’e Dene First Nation 2020).
Geographical Context	**AMA**: South/Atlantic; Park near populated zone	Point Pelee NP is bordered by several communities including Leamington, ON (pop. 27,595 (2016)) and roughly 65 km from Detroit, MI or 350 km from Toronto, ON.
	**CMB**: North/West; remote location	Tuktut Nogait NP’s nearest community is Paulatuk (pop. 265) which is serviced by only one airline from Inuvik, NWT where the Parks Canada field unit is based.

**Table 4:** Illustrative examples of differences between agreements according to governance parameters of the typology

Governance parameter	Difference	Illustrative example
# of Indigenous Groups Represented	**ABA:** Flexible Indigenous representation	The Riding Mountain Forum Agreement (2006) comprises of a Coalition of seven First Nations from Treaty 2, 4, and 1 represented by respective Chiefs but recognizes that First Nation membership may fluctuate
	**CMB:** Static	The IIBA for Auyuittuq, Quttinirpaaq, and Sirmilik NPs (1999) is between the Nunavut Inuit as represented by the Qikiqtani Inuit Association (QIA). No provisions to withdraw or terminate agreement.
	**CMB:** Member Withdrawal	The Maa-nulth Side Treaty (2012) allows individual Maa-nulth First Nations to withdraw.
Legal Strength	**RBA:** Not legally binding	The Memorandum of Understanding between PCA and Saugeen Ojibway First Nation (2021) is not a legally-binding contract but instead represents a mutual commitment to cooperation.
	**CMB:** Legally binding	Ivvavik NP is cooperatively managed according to the legal requirements of provisions in the Inuvialuit Final Agreement (1984), similar to many other CMBs.
	**CMA:** Legally binding	The Thaidene Nëné Establishment Agreement is a legal contractual agreement where the park will remain under “reserve” status until Akaitcho self-governance negotiations and land claims with Northwest Territory Métis Nation are settled (Łutsel K’e Dene First Nation 2020; Parks Canada 2020)
Governance Body Composition	**RBA**: No advisory body	The Mi’kmaq-Parks Canada Interim Arrangement (2017), among other objectives, requires Parks Canada to consult with 13 Mi’kmaq Chiefs and Councils, and commits to negotiation of terms of reference for a future advisory committee
	**ABA:** Indigenous appointees outnumber Parks Canada’s	The Terms of Reference for Point Pelee National Park First Nations Advisory Circle (2011) requires First Nation representation of up to 3 appointees from both Walpole Island First Nation and Caldwell First Nation. Parks Canada representatives: the Superintendent and two others designated by the superintendent.
	**CMB:** No Defined Body Composition	VFGN Final Agreement (1993) lists parties to be involved in cooperative management but does not define an advisory body structure
	**CMB**: Parks Canada appointees outnumber Indigenous appointees	The Wapusk NP cooperative management board consists of 10 members: 2 from Canada (including Superintendent who will have non-voting powers); 2 from Manitoba’s provincial government; 2 from both Fox Lake and York Factory First Nations appointed by Federal Minister upon FN’s recommendation; and 2 from Churchill’s local government district (Federal-Provincial Memorandum of Agreement for Wapusk National Park 1996)
	**CMB**: Indigenous appointees outnumber Parks Canada’s	In Tongait KakKasuangita SilakKijapvinga/Torngat Mountains NPR, agreements are stipulated in a way that has allowed the cooperative management board to be entirely seated by Indigenous appointees representing both Parks Canada and the Indigenous government (Youdelis 2023). In contrast, Kluane NP and NPR is cooperatively managed by a board, which according to the overlapping CAFN Final Agreement (1993) and KFN Final Agreement (2004), is seated by 2 appointees from each First Nation and only 2 from Parks Canada where the superintendent is an additional non-voting 3^rd^ member.
	**CMB:** Greater or equal Indigenous representation with independent chair or non-voting senior official(s)	The cooperative management board of Tuktut Nogait NP is equally appointed with a fifth jointly appointed chair (Tuktut Nogait Agreement, 1996)
	**CMA:** Greater or equal Indigenous representation with independent chair or non-voting senior official(s)	The Thaidene Nëné Xá Dá Yáłti (operational management board) has an equal ratio of Lutsel K’e Dene appointees to jointly appointed members by GNWT and Parks Canada, and a seventh independent chair jointly appointed by all parties (Łutsel K’e Dene First Nation 2020, p. 12; Youdelis 2023) The Nahʔą Dehé Consensus Team is comprised of 4 Indigenous representatives (representing the two Indigenous parties) and 3 individuals appointed by Parks Canada. The Chief of NDDB, the Grand Chief of DFN, and the Park Superintendent are ex-officio non-voting members (Ndahecho Gondié Gháádé Agreement 2022)
Role of Appointees/Members	**ABA:** to Represent Interest of Parties	Riding Mountain Forum Agreement (2006) is specifically designed to represent the interests of all First Nations
	**CMA:** to represent public interest	Nahʔą Dehé Consensus Team appointees are to act in the best interests of the park (Ndahecho Gondié Gháádé Agreement 2022)
Mandate	**ABA:** Recommendations based on consensus discussion provided to Minister	The TOR for Point Pelee’s First Nations Advisory Circle (2011) requires that a consensus approach that respects Indigenous cultural practices be taken. Decisions of the board are ultimately deemed as advice to the Minister for approval.
	**CMB:** Recommendations based on simple vote or consensus provided to Minister	The cooperative management board of Wapusk NP makes its decisions through simple majority vote (Federal-Provincial Memorandum of Agreement for Wapusk National Park 1996)
	**CMB:** Recommendations based on consensus are provided to Indigenous Leadership and Minister	The cooperative management board refers all matters of Tongait KakKasuangita SilakKijapvinga/Torngat Mountains NPR management, asides those that concern Inuit rights, to Indigenous and Parks Canada authorities who enact day-to-day operational decisions (Youdelis 2023)
	**CMA:** Recommendations based on consensus provided to Indigenous leadership and Minister	The Gwaii Haanas Archipelago Management Board “will strive in a constructive and co-operative manner to achieve a consensus decision of the members, which will be deemed recommendation both to the Government of Canada and the Council of the Haida Nation” (Gwaii Haanas Agreement 1993, p. 4)
Dispute Resolution	**ABA:** Dispute resolution through consensus process	The Riding Mountain Forum Agreement (2006) requires the Superintendent and Chief representing the Coalition to enter into discussions as a first stage of dispute resolution, and if a resolution cannot be found a mutually decided facilitator or mediator must be engaged for the second stage. A final third stage requires discussion between senior representatives of the parties before a mutual decision is made to continue the agreement or dissolve the agreement by mutual decision. In contrast, the TOR for Point Pelee’s First Nations Advisory Circle (2011) does not include any direction for dispute resolution.
	**CMB:** Legal Dispute Resolution Mechanisms	The IIBA for Qausuittuq NP (2015) requires parties to discuss disputes in good faith, then through mediation or legal arbitration in case of continued disagreement. Legal external recourse is not precluded. In contrast, under the Maa-nulth Side Treaty (2012), disputes concerning Pacific Rim NPR cannot be resolved by arbitration under land claim provisions but are limited to a lower stage of “collaborative negotiations” based on the expressed desires to resolve issues through discussion.
	**CMA:** Dispute resolution through consensus process	Ndahecho Gondié Gháádé Agreement (2022) distinguishes between disputes which must go through standard conduits of mediation and arbitration, and issues, which are to be formally raised and resolved through good faith discussion between parties. If parties fail to resolve issue, it will be held in abeyance and will be referred to Nahʔą Dehé Chief, Grand Chief of Dehcho First Nations and the Minister for good faith discussions. Final disagreements do not affect existing rights, jurisdiction or final authorities of any party.
Processes for Adaptation	**RBA:** Amendments made through mutual written consent, option to unilaterally terminate agreement, frequent agreement review	The Memorandum of Understanding between PCA and Saugeen Ojibway First Nation for Bruce NP and Fathom Five NMP (2021) can be amended with mutual consent at any time. Either party may withdraw from the agreement with prior written notice. The agreement has a yearly progress review between Parties.
	**CMB:** Inconsistent processes for amendments, termination, review, and implementation monitoring	Under the VGFN Final Agreement, parties must make amendments to the agreement through the UFA’s amendment process which involves Canada represented by Governor in Council and the Yukon Assembly of First Nations. In contrast, parties to IIBAs under the NLCA may amend occur directly through written consent of the parties, like in Aulavik NP (An Agreement for the Establishment of a National Park on Banks Island, 1992) The Maa-nulth Side Agreement (2012) allows for any party to terminate or withdraw from the agreement. In contrast, the IIBA for Qausuittuq NP (2015) does not provide provisions for termination or terms for withdrawal. IIBA for Ukkusiksalik NP (2001) requires an annual implementation review, and a five-year interval Agreement review. Meanwhile the IFA does not have a provision for reviewing agreement or implementation in Ivvavik NP (1982).
	**CMA:** Inconsistent processes for amendments, termination, review, and implementation monitoring	The Gwaii Haanas Agreement (1993) is to continue in perpetuity but may be dissolved by request of either party upon 6 months written notice, whereas the Tallurutiup Imanga NMCA IIBA (2019) must renegotiate a successor IIBA every seven years, unless it is terminated by mutual written consent. The Ndahecho Gondié Gháádé Agreement (2022) does not include any provisions for termination of agreement.

It is important to recognize that the focus on power-sharing aspects of the agreements ignores other dimensions of co-management which can serve as mechanisms to facilitate specific processes of reconciliation. Finegan (2018) sheds light on many of these, including employment opportunities, capacity-building initiatives, and stewardship programs. Although not a focus of this study, the typology suggests that certain agreements, for instance RBAs and ABAs, may serve an important role in renewing relationships. This is because they are negotiated, in part or in full, with this as their intent and are typically more flexible and adaptable to evolving Indigenous priorities.

There are additional constraints to the typology approach we took that need to be pointed out to understand the rationale behind the relatively narrow scope of the study. In essence, the typology does not reflect the full story of negotiated agreements as a governance institution across the national park system. For one, agreements are difficult to procure. Confidentiality clauses in agreements often limit public distribution or result in the redaction of sensitive information in public facing versions. Access to some agreements is limited to the negotiating parties alone. As well, the narrow focus on agreements excludes co-management arrangements such as the Jasper Indigenous Forum that is not protected by any agreement but serves the same role as the forums and advisory circles reviewed in this study (Johnston and Mason 2020).

Moreover, the typology fails to account for various parameters that affect governance arrangements. Typologies are a flawed approach to governance analysis due to their inherent theoretical assumption that co-management institutions can be classified through a valid and reliable system (Smith 2002). For example, there was considerable variation in the granularity and clarity of processes concerning Ministerial involvement, especially between earlier agreements (Vuntut NP) and later agreements (Qausuittuq NP). Perhaps most evident was the lack of attention in agreements to the use of Indigenous knowledge in decision-making. The lack of consistency within agreements made it difficult to make meaningful comparisons on the use of Indigenous knowledge between agreement types. In any case, as argued by Finegan (2018, p. 11), while Indigenous knowledge integration could be part of reconciliation processes, “it does not resolve underlying settler-colonial power structures”.

A final limitation of our focus on negotiated agreements is the discounting of the dynamic and adaptive relationship-building dimensions of co-management and how they affect, and are affected by, existing power structures (Carlsson and Berkes 2005; Fischer et al. 2014). Co-management may encourage Indigenous autonomy and co-governance in unanticipated ways (Feit 2005). As well, the same co-management agreement may have different implementation outcomes depending on the influence of legal and constitutional tools available to, and the capacity of, Indigenous groups across different contexts (Mulrennan and Scott 2005; Papillon 2008). Similarly, the agency of Indigenous communities and their expectations relative to those of Parks Canada in relation to co-management negotiation are not captured (Clark and Joe-Strack 2017; Parsons et al. 2021). This study calls for closer attention to place-based narratives of co-management in national parks to better understand their evolution over time. Meanwhile, the typology offers a tool to track the proliferation of shared governance approaches more accurately across the diverse contexts where national parks and NMCAs exist. This will be increasingly important as Canada advances its commitment to expand the national park, national park reserve and NMCA network in full collaboration with Indigenous partners (Canada 2021b).

## Results

The following results present a typology of co-management agreement types under Parks Canada as distinguished by their context and governance arrangements.

### The Four Types of Co-Management Agreements

The four types of co-management agreements identified in the agreement scan, based on their power-sharing structures, can be described as follows:

1. Relationship-Building Agreements (RBAs) do not create co-management structures but establish mutual commitments to collaborate in management. Examples of this type of agreement include Memorandum of Understanding (MOU) or Memorandum of Cooperation.
2. Interest-based Advisory Body Agreements (ABAs) create formal advisory structures (e.g., forums, advisory circles) to facilitate interest-based engagement of Indigenous groups. Examples include MOUs or Terms of Reference (TORs).
3. Cooperative Management Board Agreements (CMBs) create cooperative management boards with Indigenous groups which provide management recommendations to the Minister. Examples include establishment agreements, land claims and associated agreements (e.g., Impact and Benefit Agreements (IBAs^7^)).
4. Consensus Management Boards (CMAs) refer to a cooperative management board where delegated authority allows decisions of the governance body to be considered as recommendations to both authorities who can then enact decisions. Examples include establishment agreements, IBAs, and shared governance agreements.

### Key Differences Across Context and Governance Dimensions between Agreement Types

#### Context

The agreement types reveal a strict correlation with contextual parameters, as opposed to a spectrum. Accordingly, RBAs and ABAs are implemented in southern parks in, often disputed, historic treaty contexts and where Indigenous peoples were not engaged or were displaced in the establishment of the park. In contrast, CMBs and CMAs are implemented along the West Coast and in the North under the provisions of land claims and self-governance negotiations and settlements which may apply to both existing parks and parks co-established with Indigenous partners. Table 3 presents key illustrative examples of agreements across contextual parameters. While all agreements have been implemented in post-agreement contexts, similarities and differences in early involvement are distinguished through comparison of ABA agreements in Riding Mountain NP^8^, with examples of CMBs in both establishment and post-establishment contexts (Vuntut NP and Auyuittuq, Quttinirpaaq and Sirmilik NPs), and CMAs implemented post-park establishment in Nahʔą Dehé/Nahanni NPR. The contrast between agreements in historic treaty contexts in the north and south is illustrated by Bruce Peninsula NP and Thaidene Nëné NPR. The diversity of legal contexts for CMAs is demonstrated by Gwaii Haanas NPR, and Thaidene Nëné NPR. While CMB agreements predominantly exist in comprehensive land claim contexts, exceptions occur where specific claims have been settled by court decisions, as in Wapusk NP or in Pacific Rim NPR where co-management was established through a cooperative agreement (2006) prior to the ratification of the Maa-nulth Final Agreement in 2011. Similar relationships between geographical context, specifically in terms of region and remoteness, and agreement type are illustrated through two extreme cases of AMAs and CMBs (Point Pelee NP and Tuktut Nogait NP).

#### Governance

The spectrum from limited power sharing in RBA agreements to significant power sharing in CMA agreements reflects differences in governance subparameters relating to Indigenous representation, legal strength of agreements, governance body composition and mandates, and processes for dispute resolution and adaptation. The typology suggests that CMBs have a high degree of variability across certain governance parameters, which is captured in Table 4 along with other key illustrative examples of agreements across governance parameters.

We use Riding Mountain NP, Auyuittuq, Quttinirpaaq and Sirmilik NPs, and Pacific Rim NPR to illustrate the greater flexibility of Indigenous membership in RBA and ABA agreements than those observed in CMBs and CMAs. These illustrations are fundamentally tied to the legal strength of agreements (either non-binding in RBAs and some ABAs or binding in CMBs and CMAs) which is captured in examples from Bruce NP (RBA), Ivvavik NP (CMB), and Thaidene Nëné NPR (CMA).

The spectrum of Indigenous to Crown representation across governance bodies ranges from undefined to equal or greater Indigenous representation. RBAs, like the Mi’kmaq-Parks Canada Interim Arrangement, do not establish governance structures, whereas ABAs create advisory structures (e.g. forums) resembling cooperative management boards, where Indigenous representation may outnumber state government representation, as illustrated by the Point Pelee NP First Nation Advisory Circle. Inconsistencies in the governance body composition and Indigenous representation of cooperative management boards of CMBs is highlighted through a diversity of examples from Vuntut NP, Wapusk NP, Tongait KakKasuangita SilakKijapvinga/Torngat Mountains NPR, and Kluane NP and NPR. Appointees to advisory bodies may act in the public interest or in the interests of parties, depending on agreement type, as highlighted by the Riding Mountain Forum Agreement (2006) (ABA) and the Nahʔą Dehé/Nahanni NPR agreement (2022) (CMA). Additionally, we note that neither CMBs nor CMAs are categorically “politically independent bodies”, according to the scan. The advisory bodies of CMAs and CMBs may require the joint appointment of an independent chair or non-voting senior representatives (i.e., Chief and Superintendent). In other CMBs, the park superintendent is an ex-officio member of the governance body as a nonvoting observer, such as in Kluane NP and NPR. This is in contrast to the other CMBs of Tuktut Nogait NPR and CMAs like Thaidene Nëné Establish Agreement which elect a jointly appointed chair, and CMAs such as those of Gwaii Haanas NPR and Nahʔą Dehé/Nahanni NPR, where both the FUS and Indigenous leadership (e.g., Chief) are present as nonvoting observers.

For the majority of CMAs, ABAs, and CMBs, “decisions” made between parties are deemed as advice recommended to the Minister for final approval. The Minister then chooses to accept, vary, or set aside and replace recommendations. The decisions of CMB boards are made by simple majority vote or in some cases by consensus, as shown in the Wapusk NP example. ABAs may also stipulate how advice or decisions are to be produced through consensus discussions between parties, as exemplified in the Terms of Reference for Point Pelee National Park First Nations Advisory Circle (2011). Similarly, CMAs are distinguished through their stated or implied governance authorities which are enabled through consensus models which allow parties to make and implement decisions without Ministerial involvement (Gwaii Haanas Agreement 1993). In special cases of CMBs, the cooperative management board may refer recommendations about select operation and management matters to both the Superintendent and Indigenous leadership allowing decision-making to occur at the park level if parties agree to implement a resolution. The only example where this was identified was in Tongait KakKasuangita SilakKijapvinga/Torngat Mountains NPR, where only disputes or decisions affecting Inuit rights are necessarily provided to the Minister (Youdelis 2023).

Across the spectrum of agreements, dispute resolution mechanisms range from wholly absent to defined consensus resolution protocols backed by legal resolution mechanisms. ABAs may or may not include protocols for dispute resolution, as illustrated by the Riding Mountain NP and Point Pelee NP agreement examples. Qausuittuq NP is used as a key example of a step-wise dispute resolution process under CMBs which typically finalize in legal arbitration (2015). This is in contrast with the inability of Maa-nulth First Nations to enter into legal arbitration through the Maa-Nulth Side Treaty (2012). These processes are compared with those of CMAs, in which matters are typically put into abeyance and entail an escalation of consensus-driven discussions between senior representatives (e.g. Chief and Minister), possibly with the aid of a mutually agreed-upon third-party mediator or arbitrator (e.g. Ndahecho Gondié Gháádé Agreement 2022).

Processes for adaptation revealed the greatest variability across agreements and within agreements, especially for CMBs and CMAs. For example, RBAs such as in Bruce NP provide significant flexibility for parties to amend or terminate the agreement and require frequent agreement reviews, as a more or less consistent practice. However, Gwaii Haanas NPR, Tallurutiup Imanga NMCA, and Nahʔą Dehé/Nahanni NPR agreement examples illustrate the range of differences within the CMA type. Within the CMB agreement type, amendments can be made through written consent between parties or require additional steps through external claims-based mechanisms, as illustrated by Vuntut NP and the Inuit IBAs. These may or may not include periodic agreement reviews as illustrated by Ukkusiksalik NP and Vuntut NP. Likewise, the availability of provisions for termination of the agreement varies between CMBs, as contrasted by Pacific Rim NPR and Qausuittuq NP.

### Implications for Shared Governance

Using the key differences of context and governance arrangements between the different co-management agreement types, as delineated by the agreement typology, we can explore how these agreements measure up to international standards of shared governance options presented in Table 5 (Milko 2020). Co-management must be understood as a governance institution operating at both constitutional and management levels (Petursson and Kristofersson 2021). This requires clarification of Canadian national parks legislation and policy. First and foremost, the Minister responsible for national parks and NMCAs cannot restrict their authorities in entering into cooperative agreements (Langdon et al. 2010). According to the CNPA (2000)^9^^,^^10^, in the absence of agreements with Indigenous governments, decision-making powers and responsibilities for administration and management of a national park revert to the Minister’s discretion. Furthermore, while the CNPA (2000) is not to derogate or abrogate from Aboriginal or treaty rights as protected by the *Constitution Act*, 1982, the Minister is entitled to make regulations regarding harvesting and permitting.

**Table 5:** Four shared governance options for national parks according to power-sharing dimensions (Milko 2020)

	State Governed and Managed	State governed and managed with an Indigenous advisory body to advise on /participate in management	State governed and managed with an indigenous advisory body to advise on governance and management	Jointly Governed, Jointly Managed
Power sharing				
Decision making level and control	Decision-making agency controlled; Indigenous input may be provided through consultation	Decision-making Agency controlled; Indigenous input may be provided on a local scale	Decision-making Agency controlled; Indigenous input may be provided via agreed structures	Decision making defined by both Indigenous law and culture, and partner requirements - codified in jointly developed agreement or legislation.
Rules Definition	Rules defined by agency and constrained only by legally enforced Indigenous rights	Rules defined by Agency as constrained by legislative and policy recognition of Indigenous rights	Rules defined by Agency as constrained by constitutional, legislative, and policy recognition of Indigenous Rights	Rules defined by Agency and Indigenous peoples.

With this in mind, Parks Canada retains governance powers across the majority of national parks and NMCAs, according to the scope and proportion of agreement types. It could even be argued that advisory bodies for management do not influence governance approaches to any significant degree, as Parks Canada has a legal duty to consult Indigenous groups on planning and management in all protected areas. This is in accordance with both the Act (CNPA 12 (1)) and Supreme Court of Canada jurisprudence of the early 2000s that clarified the fiduciary duty of the Crown, such as in *Haida Nation v. British Columbia (Minister of Forests)* (2004) (Brideau 2019). Of course, co-management institutions under constitutionally protected land claims imply a more fulsome recognition of Indigenous rights in park management (Canada 2014). In exceptional cases involving CMBs, Indigenous advisory bodies provide advice on governance and, to a certain extent, facilitate joint decision-making. This is contingent upon the structures through which Indigenous authorities are engaged in receiving and endorsing recommendations from the cooperative management boards (e.g., Tongait KakKasuangita SilakKijapvinga/Torngat Mountains NPR). It is also possible that the presence of amendment and review processes offer opportunities for CMBs to be altered and evolve towards more equitable shared governance arrangements.

The typology indicates that consensus-based management agreements are supportive of joint governance and joint management arrangements but remain constrained by Canadian law. Indeed, no agreements have reflected the approach taken by the Haida Nation and Parks Canada to “agree to disagree” on the issues of sovereignty, jurisdiction, and title over Gwaii Haanas (Gwaii Haanas Agreement 1993). Thus, Indigenous jurisdiction within national parks and national park reserves is recognized insofar as it is recognized in land claims. Only through Aboriginal claims and treaty processes have Indigenous groups been able to negotiate and enter into cooperative management agreements that do not limit the rights, jurisdiction, authority, obligations, or responsibilities of *both* parties (Gwaii Haanas Agreement 1993). It is therefore reassuring that these principles are reinforced by rigorous dispute resolution mechanisms involving both legal recourse and consensus resolution between senior authority.

### Co-Management for IPCA Establishment: A New Type of Co-Management Agreement?

While the Indigenous-Crown relationship as expressed in the Gwaii Haanas Agreement remains anomalous in the scope of co-management arrangements extant under Parks Canada, collaborations within the past five years and the rise in IPCA establishment suggest a sea change concurrent with commitments towards reconciliation. It is important to recognize that the shared governance typology does not encompass all possible variants of Indigenous-led shared governance types as described in Lyver et al. (2014) (e.g., Indigenous governed, jointly managed). In light of the present legislative framework, all national parks are viewed as state governed according to Canada’s perspective. A recent exception could be Thaidene Nëné, where LKDFN designated Thaidene Nëné as an Indigenous Protected Area under Dene laws but chose to utilize an array of state legislation to protect certain portions of the overall area, including the CNPA (2000). However, the typology fails to capture the complexity of Indigenous-led, multi-tiered governance structures such as those in Thaidene Nëné where certain governance and management matters are under the shared responsibility of the xá dá yáłtı and a separate regional consensus-based management board seated by appointees from other neighboring First Nations, Parks Canada, and the territorial government (established through a separate TOR) (LKDFN and NWT 2019). As this is the only current example of such an arrangement, it is unclear whether this represents a new type in our agreement typology. Indeed, while the Establishment Agreement between LKDFN and Parks Canada remains confidential, there is no reason to assume that the portion of the area under national park reserve status is exempt from the Canadian National Parks Act. This assumption is supported by the establishment agreement which implies that, while decision-making will be led by xá dá yáłtı, on which Parks Canada will have an appointed member, another separate management board will be established for the NPR itself (LKDFN and NWT 2019). However, it is promising that the tension between Indigenous and Parks Canada governance approaches is openly acknowledged in one of the protected area’s establishment agreements which states that the parties will “seek to manage Thaidene Nëné in a manner that is consistent with the management of the National Park Reserve while respecting the differences between the National Park Reserve and Thaidene Nëné” (LKDFN and NWT 2019, p. 15). While this phrasing does not exactly mirror the language incorporated into the 1993 Gwaii Haanas Agreement concerning parallel sovereignties and jurisdiction, and despite the fact that the underlying Canadian laws and policies regarding Indigenous self-determination have not changed, Indigenous authorities creating IPCAs (or comparable entities) may now have an opportunity to contemplate similar partnerships with Parks Canada in pursuit of their own aspirations and priorities.

Finally, the Thaidene Nëné example reveals an additional challenge for Parks Canada in achieving shared governance across the national park system. Recent territorial legislation has enabled the NWT government to engage in governance and management agreements with Indigenous governments and organizations, offering unique collaborative opportunities for Indigenous-led conservation that are not currently present in other jurisdictions (NWT 2019). This development may imply that such collaborations will persist in creating governance disparities across the national park system. While it is too early to make definitive conclusions regarding the impact of Thaidene Nëné for Parks Canada’s co-management framework without further research, it is evident that such agreements represent a significant effort in re-purposing existing mechanisms to support Indigenous-led conservation efforts.

## Discussion

The disaggregation of co-management agreements using the parameters of the typology outlined above offers a detailed portrayal of co-management as a governance institution within the national park context. This typology establishes a framework grounded in a comprehensive database, which facilitates comparative analysis of negotiated agreements. For instance, it highlights how Canada’s constitutionally-protected Indigenous rights framework results in an uneven playing field for Indigenous groups participating in co-management (Nikolakis and Hotte 2019). Indeed, the recognized roles in governance and management granted to northern and western Indigenous groups through co-management agreements have yet to be observed in eastern and southern contexts. This disparity is further compounded by the differences in adaptation processes, which may lead to the establishment of robust power-sharing arrangements in only certain parks.

The typology also facilitates a deeper comparative exploration of mechanisms (e.g., board composition, dispute resolution) within and between agreement types for their potential to support Indigenous governance and self-determination. On the one hand, the typology suggests that negotiated agreements alone do not create secure power-sharing arrangements that facilitate Indigenous governance or self-determination in southern parks and within southern treaty contexts (Dearden and Bennett 2016). While this claim may appear contradicted by the examples of Wapusk NP (CMB) and Thaidene Nëné NPR (CMA), both situated in historic treaty areas (typical of southern regions of Canada), there were active claims or ongoing rights negotiations during the establishment of those parks. On the other hand, examples of consensus models demonstrate that co-management is not necessarily incompatible with Indigenous governance and self-determination (Gray and Kuokkanen 2020). Rather, these agreements indicate that national park or NMCA designation could potentially bolster Indigenous self-determination and resurgence, provided that certain conditions are met (Artelle et al. 2019; Zurba et al. 2019). As Smith (2020) asserts, co-management relationships must be rooted in self-determination or true nation-to-nation relationships to enable shared governance and serve as a vehicle to support reconciliation. In modern treaty or self-governance contexts where Indigenous jurisdictions are recognized, co-management may be supportive of constitutional or political reconciliation and create a platform for engaging Indigenous laws and value systems (Martin 2016; Turner 2013). For better or for worse, a cursory chronological assessment of the typology suggests that consensus-based governance models are becoming increasingly common, likely inspired and shaped by the governance institutions created in earlier agreements. These appear likely to replace more conventional cooperative management boards in modern treaty contexts (e.g. Tallurutiup Imanga NMCA) (Petursson and Kristofersson 2021; Thomlinson and Crouch 2012).

While co-management can facilitate the reconciliation of Indigenous and non-Indigenous interests and worldviews, fostering mutual respect (Nesbitt 2016), such institutions alone do not address underlying settler-colonial power structures (Finegan 2018). According to Clark and Joe-Strack, even “at its fullest expression, co-management is still only a part of what’s required to realize the vision of self-determination that claim agreements were intended to move society towards” (2017, p. 73). More importantly, emergent governance models, like that of Thaidene Nëné, that are supportive of Indigenous self-determination and self-governance appear to be more reflective of the capacity of Indigenous nations themselves (e.g. Thaidene Nëné), as opposed to the state’s willingness to devolve its authority (Łutsel K’e Dene First Nation 2020; Thomlinson and Crouch 2012). Similarly, while the Gwaii Haanas NPR and Pacific Rim NPR agreements were initiated without settled claims, their negotiation processes resembled those of land-claims, necessitating a sustained and substantial negotiating capacity on the part of the participating Indigenous groups (Alcantara 2013; Timko and Satterfield 2008). Moreover, as a federal agency, Parks Canada’s ability to honor Canada's legal commitments to Indigenous peoples, especially in southern parks, is dependent upon overarching treaty processes, a constraint reflected in the typology through the influence of contextual parameters (i.e., treaty and geographical context) and temporal patterns across agreement types (Thomlinson and Crouch 2012; Langdon et al. 2010). Indeed, at a policy level, the CNPA has not yet formally established co-management power-sharing principles. Consequently, a uniform and equitable approach to collaborating with Indigenous groups has not been consistently implemented (Sandlos 2014; Thomlinson and Crouch 2012). However, there is a Ministerial commitment indicating that an Indigenous stewardship policy may be in the works to address this gap (Parks Canada Agency 2021). The recent policy on NMCA establishment and management, which gives primacy to the role of Indigenous communities in stewardship, may be early evidence of changes to come (Parks Canada Agency 2022a). Regardless, efforts to advance state commitments in creating new national parks without corresponding adjustments to Canadian legislation, coupled with a transparent, deliberate policy framework for Parks Canada to engage in shared governance across all national parks and other protected areas under their scope, could potentially restrict Canada’s ability to fully realize its vision for reconciliation.

An alternative perspective offered by Nesbitt (2016) asserts that adjustments to legislation or land claims processes may not be necessary since consensus-based decision making is not necessarily at odds with conventional advisory CMBs if Indigenous and state authority is meaningfully represented at the table. Likewise, Snook et al. (2018, p. 68) argue that, when contemplating the intent of co-management agreements, “the focus should not be placed on meeting the minimum legal requirements laid out in the original land claims documents; rather, these documents can be viewed as the minimum baseline from which to build all future decisions and actions”. This position is not without support. For example, decisions of advisory co-management boards have been rarely referred to or overturned by the Minister (Nesbitt 2016; Youdelis 2023). In instances where decision-making is referred to the Minister or their designate, the exhaustive protocols for decision-making and dispute resolution observed in more recent co-management agreements may serve in bringing clarity to the persistent issue of Ministerial authority. In addition, while not captured by the typology presented above, there is precedence within co-management agreements for recommendations concerning the suitability of an appointee to a cooperative management board to be based on their connections to the land and role in the community. This has led to an all-Indigenous cooperative management board in both Tongait KakKasuangita SilakKijapvinga/Torngat Mountains NPR and Thaidene Nëné’s Xá Dá Yáłtı (Youdelis 2023). However, this type of de-facto self-governance is at least partly enabled by the higher proportions of Indigenous peoples in the North, a condition that is unlikely to arise in southern contexts with more diverse demographics and stakeholder interests (Atkinson 2001). Under these unique agreements, board members are required to act in the best interests of the land, as opposed to representing their appointing parties. While this approach may better reflect a shared vision for land stewardship, such arrangements may be a limitation as board independence has historically been a source of conflict within boards and between boards and federal government (White 2018).

## Conclusion

While the typology framework presented in this paper overlooks parameters that reflect Indigenous interests and priorities, the study confirms that co-management agreements are strongly connected to contextual factors, such as the treaty context. The spatial and temporal patterns of agreement distribution, as revealed by the typology, indicate that Parks Canada has advanced co-management agreements in accordance with contextual factors, such as legal and policy constraints, which seem to indicate that the agency is limited in its ability to respond to the full spectrum of Indigenous demands due to external factors. This approach to Indigenous engagement has inevitably led to disparities across national parks in the extent to which Indigenous groups are involved in governance, as only specific types of co-management agreements allow Indigenous groups to meaningfully participate in decision-making according to their own legal orders and knowledge systems, such as through consensus-based management bodies.

We acknowledge that this study provides only a partial explanation of the factors that may constrain Parks Canada’s willingness and capacity to re-imagine itself. As the agency continues on its path to reconciliation, further research is needed to better understand the evolution of Parks Canada’s organizational culture with regards to the balancing of competing rights and uses of parks. Due to political and economic dynamics at various scales, the philosophical motivations underpinning national parks has shifted over time, fluctuating from notions of nationalism, preservation, and the ideal of untouched wilderness to venues for economic growth, tourism, and resource exploitation (Mortimer-Sandilands 2009; Searle 2000). This diversity is also spatially evident, reflected in the differences in levels of Indigenous leadership and expectations regarding collaboration between national parks in the North and the South (Canada 2001; Johnston and Mason 2021; Nesbitt 2016; Notzke 1995). As underlined by Youdelis (2016) and Youdelis et al. (2020), Western philosophical values and biases, which continue to dominate conservation practices and organizational culture in national parks, are credible threats to meaningful Indigenous engagement.

Indigenous leaders and scholars have argued that making space for Indigenous forms of conservation, rooted in Indigenous systems of governance, legal orders, and knowledge systems will ultimately require a fundamental restructuring or (re)-Indigenization of the current conservation paradigm (Hessami et al. 2021; M’sɨt No’kmaq et al. 2021). The extent to which emergent and revised models of co-management – identified in the typology – particularly when paired with engagement framework, such as ethical space and two-eyed seeing, can support such a renovation has yet to be determined.

As IPCA-supportive and consensus co-management models emerge, they offer the prospect of a new era of Indigenous participation in national park governance and management. However, there is a danger of accepting a better set of Indigenous-state relationships based in a comparison to historical benchmarks that have set a low bar (Sandlos 2014). We instead echo calls for attention to the ongoing hesitation of the Canadian government to make more fundamental adjustments to the underlying legislation to support genuine Indigenous authority and jurisdiction in national park governance (Sandlos 2014). Each Indigenous community has distinct needs and desires for reconciliation within the conservation context. Parks Canada’s responsiveness to the diversity of Indigenous priorities and aspirations as they evolve will depend on its capacity to facilitate genuine shared governance arrangements, which, in turn, hinges on Canada’s willingness to reconcile with its own claims to sovereignty.

## Footnotes

1 Canada Target 1, responding to the Aichi Biodiversity Targets, aims to increase land conservation and marine conservation by 17% and 10%, respectively. Recently, these goals were updated to protect 30% of land and water by 2030 (Canada 2022)

2 Co-management, or according to Parks Canada, “cooperative management” or joint management, refers to a diverse array of co-management approaches denoting “the sharing of power, responsibility, knowledge and decision-making” (Dearden and Bennett 2016, p. 11)

3 While there are a small number of examples of co-management agreements in National Historic Sites, some of which cover substantial land bases (e.g. Saoyú-?ehdacho NHS), they are excluded from this study. This is largely due to the disparate nature of the Historic Sites and Monuments Act (1985) relative to the Canadian National Parks and National Marine Conservation Area Acts.

4 While the focus of the study concerns these three protected area types, we frequently use national parks in a collective sense to refer to all types.

5 Secondary sources used for Torngat NP Inuit IBA and Thaidene Nëné NPR Establishment Agreement

6 Parks Canada has described three types of co-management agreements: Relationship-Building Body, Cooperative Management Board, and Consensus Management Body. We separate the first agreement type into two types: relationship-building agreement (i.e., one that creates no advisory body structure) and an interest-based advisory body agreement.

7 While Impact and Benefit Agreements refer to much broader set of agreements typically signed between Indigenous groups like the Dene, Cree, Métis, and Inuit and private industry, according to Inuit land claims, Inuit Impact and Benefit Agreements (IIBAs) are to be negotiated for the establishment of national parks.

8 For clarity of results, agreements are generally referred to herein by the associated national park or NMCA.

9 s. 8 (1), “the Minister is responsible for the administration, management and control of parks.” s. 10 (1), “the Minister may enter into agreements with federal and provincial ministers and agencies, local and aboriginal governments, bodies established under land claims agreements and other persons and organizations for carrying out the purposes of this Act.”

10 The Canada National Marine Conservation Areas Act (S.C. 2000, c. 18) mirrors the subsections of the CNPA referred to herein.
